# Music Intervention Approaches for Alzheimer’s Disease: A Review of the Literature

**DOI:** 10.3389/fnins.2019.00132

**Published:** 2019-03-12

**Authors:** Melissa Leggieri, Michael H. Thaut, Luis Fornazzari, Tom A. Schweizer, Joseph Barfett, David G. Munoz, Corinne E. Fischer

**Affiliations:** ^1^Keenan Research Centre for Biomedical Research, Li Ka Shing Knowledge Institute, St. Michael’s Hospital, Toronto, ON, Canada; ^2^Faculty of Medicine, Institute of Medical Science, University of Toronto, Toronto, ON, Canada; ^3^Music and Health Research Collaboratory, Faculty of Music, University of Toronto, Toronto, ON, Canada; ^4^Division of Neurology, Department of Medicine, University of Toronto, Toronto, ON, Canada; ^5^Division of Neurosurgery, Department of Surgery, Faculty of Medicine, University of Toronto, Toronto, ON, Canada; ^6^Division of Nuclear Medicine, Department of Medical Imaging, St. Michael’s Hospital, Toronto, ON, Canada; ^7^Department of Laboratory Medicine and Pathology, University of Toronto, Toronto, ON, Canada; ^8^Department of Psychiatry, Faculty of Medicine, University of Toronto, Toronto, ON, Canada

**Keywords:** Alzheimer’s disease, music therapy, music-based intervention, music listening, cognitive function

## Abstract

Music interventions have been widely adopted as a potential non-pharmacological therapy for patients with Alzheimer’s disease (AD) to treat cognitive and/or behavioral symptoms of the disease. In spite of the prevalence of such therapies, evidence for their effectiveness report mixed results in the literature. The purpose of this narrative review is to investigate the effectiveness of various intervention strategies (music therapy vs. music listening techniques) and music type used in the intervention (individualized vs. non-individualized music) on cognitive and behavioral outcomes for persons with AD. Databases were searched for studies using either active music therapy or music listening techniques over the last 10 years. These studies were in English, included persons with AD dementia, and whose protocol gathered pre- and post-intervention outcome measures. We initially identified 206 papers which were then reduced to 167 after removing duplicates. Further review yielded 13 papers which were extensively reviewed, resulting in a final sample of six papers. Our analysis of these papers suggested that, regardless of the music intervention approach, individualized music regimens provided the best outcomes for the patient. Furthermore, music listening may act as a relaxation technique and therefore provide a long-term impact for the patient, while active music therapy may acts to engage participants through social interaction and provide acute benefits. Our findings suggest that music techniques can be utilized in various ways to improve behavior and cognition.

## Introduction

Alzheimer’s disease (AD) is the most common form of dementia with an estimated 50 million people living with the disease today (World Health Organization, 2018). It is marked by decreased cognitive functioning (memory, visuospatial issues, and executive functioning), emotional control, and neuropsychiatric symptoms such as apathy, depression, and agitation (Lyketsos et al., 2002). Medications for AD aim to improve cognition and relieve behavioral symptoms, however, many approved drugs provide only modest benefits for the patient (Lanctôt et al., 2003; Casey et al., 2010). As a result, there has been an increase for research for non-pharmacological interventions to reduce symptom burden for AD persons and their caregivers.

In recent years, music interventions have grown in popularity as a method of non-pharmacological treatment for persons with AD for a number of reasons. First, there is evidence to suggest that music for memory can remain intact for persons with AD, even while experiencing rapid cognitive decline (Cuddy et al., 2012). This is thought to be because musical memory networks are separate from traditional temporal lobe memory networks (Platel et al., 2003; Satoh et al., 2006) which are spared until the later stages of the disease (Jacobsen et al., 2015). According to these studies, music activates a broad network in the brain rather than a single “music area.” Particularly, when listening to familiar music (such as popular folk songs, nursery rhymes, and songs on top 100 charts), musical memory retrieval involved areas both within and outside of the temporal lobes, including frontal and parietal regions (Platel et al., 2003; Satoh et al., 2006; Jacobsen et al., 2015). This diffuse network may allow sparing of musical memory functions. Furthermore, Jacobsen et al. (2015) utilized PET to investigate the degree in which music listening areas are affected by AD pathology, such as such as amyloid build up and glucose metabolism, compared to the rest of the brain and found that music listening areas experienced less pathology. The ability for persons with remember music makes music a unique stimuli which effectively engages persons with AD.

Another reason why music interventions are becoming popular with this population is because behavioral studies have shown that music can improve some cognitive functions in AD persons. For example, music in the background has been shown to improve autobiographical recall (Foster and Valentine, 2001; Irish et al., 2006; El Haj et al., 2012b). According to Moscovitch (1992), “involuntary memories” are memories which can be retrieved automatically by a cue. El Haj et al. (2012a) believe that memories evoked by music contain the same properties as involuntary memories. In other words, music can be used as a cue to evoke involuntary autobiographical memories which are specific and invoke an emotional response. Furthermore, Simmons-Stern et al. (2010, 2012) found than music enhanced verbal encoding of information. The ability of music to enhance encoding, memory and cognition in AD persons has been attributed to modulating physiological responses. It has been postulated that music’s ability to induce arousal and evoke positive emotional responses can activate the parasympathetic or sympathetic nervous system, depending on the type of music and rhythm, to in turn alleviate neuropsychological symptoms and enhance encoding efforts (Peck et al., 2016). One study by de la Rubia Ortí et al. (2018) provided evidence that music can improve emotional state in persons with AD lowering stress levels as measured by cortisol in saliva. Music interventions have also been shown to have other positive physiological effects on AD persons that may affect cognition and behavior, such as improving sleep by increasing melatonin levels (Kumar et al., 1999) and balancing hormones without the adverse effects of hormone replacement therapy (Fukui et al., 2012).

Lastly, music interventions have gained an increasing amount of interest in researchers and caregivers because, conceptually, it is an inexpensive, easily implemented, and highly enjoyable means of treatment for persons with AD. Behavioral studies investigating music interventions in AD report of low drop-out rates and high engagement in those with the disease (Guétin et al., 2009; Arroyo-Anlló et al., 2013; Sakamoto et al., 2013; Li et al., 2015; Giovagnoli et al., 2017; Gómez Gallego and Gómez García, 2017).

Music is highly versatile and accessible, which allows it to be used in patient populations in a variety of ways. Raglio and Oasi (2015) described three music approaches used in clinical settings: music therapy, music listening, and general music-based interventions. Music therapy is defined by the Canadian Association of Music Therapists (2016) as “a discipline in which credentialed professionals use music purposefully within therapeutic relationships to support development, health, and well-being.” Music therapy involves a crucial component of client/therapist interaction through an empirically supported model, and can consist of active (involving improvisation, singing, clapping, or dancing) and/or receptive (music listening purposefully to identify emotional content emerging from music) techniques (Raglio and Oasi, 2015). Music listening approaches involve a music therapist to create a music playlist for the client, which can be individualized programs or chosen by the therapist (Raglio and Oasi, 2015). Recent literature suggests that individualized music is most beneficial in AD by improving autobiographical memory (Foster and Valentine, 2001; Irish et al., 2006; García et al., 2012; Peck et al., 2016). Generalized music interventions involve the use of music without a music therapist with the goal of improving the well-being of the patient. These methods can also use active or music listening protocols. Music listening is used to “stimulate verbalization, memories, or to encourage of relaxation” (Raglio and Oasi, 2015).

Previous reviews have been published investigating the impact of music intervention on persons with dementia.Abraha et al. (2017) conducted a systematic review which summarized the results of six previously published systematic reviews on various non-pharmacological interventions. They found that interventions which included music were best at reducing behavioral symptoms of dementia. Specifically, music reduced agitation and anxiety. Another large review by van der Steen et al. (2018) found that music therapy was effective in reducing depressive and overall behavioral symptoms in their dementia participants. However, their investigation found little evidence to suggest there are benefits for anxiety, cognition, or overall quality of life. Another systematic review and meta-analysis (Tsoi et al., 2018) found that music therapy involving listening to music was more effective in reducing behavioral symptoms compared to active music therapies. Again, this study found a lack of evidence to suggest that music interventions provide benefits on cognition for persons with dementia. Contrary to these results, Fusar-Poli et al. (2017) found that active music therapy improved global cognition for persons with dementia. As well, Zhang et al. (2017) found that, after assessing for heterogeneity, music therapy had a positive outcomes for cognition for dementia.

Regardless of the mixed results in the literature, many clinicians and researchers suggest that music should be used in a medical setting (Koelsch, 2009; Kobets, 2011). In this review, we will examine the existing literature on music interventions involving individuals with AD dementia and summarize the various techniques used and their impact on cognition and behavior for this population.

## Methods

### Inclusion and Exclusion Criteria

For this review, we included studies published in the last 10 years (2008–2018), and available in English with pre- and post-intervention data collection in cognitive and/or behavioral domains. The intervention must meet the definition as either music therapy, music listening or generalized music-based interventions (active or music listening), and can be individualized or non-individualized (Raglio and Oasi, 2015). Generalized music approaches without a music therapist must be validated by a caregiver or conducted in a controlled setting to ensure adherence to protocol. The studies gathered in our review included only patients with AD dementia. Studies that were excluded included reviews, letters to the editors, studies which did not involve a music intervention or included an intervention other than music approaches, studies using a mixed intervention strategy, or studies that included a diagnosis of dementia other than AD, such as vascular dementia, Lewy body dementia, or mixed dementia.

### Search Strategy

The electronic databases MEDLINE, Pubmed, and PSYCHINFO were searched using the terms “AD” and “music intervention” or “music therapy” or “music based intervention.” The abstracts of all results from this search were read and sorted whether they adhered to our inclusion or exclusion criteria. These papers were then read for their entirety and further exclusions were made based on the inclusion criteria.

## Results

### Search Results

Our initial search of the databases resulted in 206 papers meeting search criteria. After removing repeated articles between the databases, 167 papers were included in the search. After reviewing titles and abstracts for meeting the criteria above, 13 papers were identified. A thorough reading of the papers resulted in size papers meeting criteria of this review (Table 1) (Guétin et al., 2009; Arroyo-Anlló et al., 2013; Sakamoto et al., 2013; Li et al., 2015; Giovagnoli et al., 2017; Gómez Gallego and Gómez García, 2017). The seven exclusions from the 13 papers identified were excluded due to: including no primary outcome of cognition or behavioral measure, non-AD dementia population included in the study, a study investigating acute effects on a small population after an 18-min live one-on-one session, and AD being assessed only as a covariate.

### Music Approaches for Studies That Met Inclusion Criteria

All studies included in this review involved an intervention classified as either an active music therapy or music listening (Raglio and Oasi, 2015). The music was gathered by the authors based on either the patient’s preferences (individualized) or chosen by the experimenter (not individualized). All the participants were diagnosed with AD dementia. Studies varied in the method of music exposure, setting, and type of music used.

Three studies included in this review implemented a music listening approach without an active component or music therapist (Guétin et al., 2009; Arroyo-Anlló et al., 2013; Li et al., 2015). These studies involved listening to music streamed to their rooms or headphones under the supervision of their caregivers. Li et al. (2015) used a non-individualized general music listening approach involving listening to classical music daily with their caregivers. Participants were instructed to listen to Mozart’s Sonata for Two Pianos in D major for 30 min in morning and Pachelbel’s Canon in D major for Violins for 30 min before sleep. Conversely, Guétin et al. (2009) and Arroyo-Anlló et al. (2013) used individualized playlists based on their participant’s interests. Arroyo-Anlló et al. (2013) compared a familiar music listening group to a non-familiar music listening group. Participants were asked to listen to their given music program and listen attentively in a quiet room with headphones and without distractions. Interestingly, the approach used by Guétin et al. (2009) used a specific method of music listening to induce relaxation, called the “U Sequence” (Guétin et al., 2005; Jaber et al., 2007), where rhythm, orchestral formation, frequency, and volume is slowly reduced, then increased again in a “re-enlivening” phase. The music was streamed via headphones to patient’s room.

Two studies investigated solely active music therapy led by at least one music therapist (Giovagnoli et al., 2017; Gómez Gallego and Gómez García, 2017). Giovagnoli et al. (2017) used active music therapy which adopted a “non-verbal approach with free sound–music interactions, using rhythmical and melodic instruments.” This involved allowing participants to choose instruments and play them freely. They were instructed to appreciate sounds and movement and to create interpersonal relationships with others and evoke emotions. The intervention was not individualized to the participant. Gómez Gallego and Gómez García (2017) used active music therapy with individualized music based on the participant’s tastes. Sessions included a welcome song (patients greeted and introduced themselves), rhythmic accompaniment (clapping hands or playing music instruments), moving to background music (moving arms and legs to music, dance therapy with hoops and balls), guessing songs, and farewell song.

One study compared outcomes between a music listening intervention group to an active music therapy group led by a team of clinicians. The study by Sakamoto et al. (2013) contained three experimental groups (music listening, active music therapy, control groups) in order to compare active music therapy vs. music listening vs. a control group. The music listening sessions involved an individualized music playlist via a CD player with no interaction with their caregivers or a music therapist. The active group also used the CD player with individualized music but the sessions were led by music therapists, occupational therapists, and nurses, who facilitated activities such as clapping, singing, and dancing. Participants in control group remained in a silent room with their caregiver.

**Table 1:** Studies investigating music interventions for AD.

Study	Participants	Music intervention length	Music intervention method	Outcome measures	Summary of Results
Giovagnoli et al. (2017) *Neurological Sciences*	Probable mild to moderate AD (MMSE > 15). (CT) (*n* = 13) vs. Active music therapy (AMT) (*n* = 13) vs. Neuro-education (NE) (*n* = 13)	12-weeks, including two 25 min group sessions per week	Active music therapy Led by music therapist Non-verbal approach with free sound–music interactions, using rhythmical and melodic instruments. Participants choose instruments and played them freely. Participants and were instructed to appreciate sounds and movement and to create interpersonal relationships with others and evoke emotions. Not individualized	Measures taken at beginning and end of intervention (Week 1 and week 12). Follow up 3 months after intervention completion *Cognitive:* Word Fluency Task (WFT) on phonemic and semantic cue Short Story Test (SST) Corsi Blocks Span Rey Complex Figure copying and delayed recall Rey Auditory Verbal Learning (RAVLT) Trail Making Attentive Matrices Weigl Sorting Raven Colored Progressive Matrices (RCPM) Street Completion tests *Behavioral:* Beck Depression Inventory (BDI) State Trait Anxiety Inventory (STAI Y-1, STAI Y-2) Lubben Social Network Scale (LSNS)	*Cognitive:* AMT showed less clinically significant improvements in WFT scores than CT (7.69% and 61.54%, respectively), but more than the NE group (none). AMT showed less clinically significant improvements in SST scores than CT (23.08% and 38.46%, respectively), but more than the NE group (none). *Behavioral:* AMT showed improvement of mood similarly to CT and NE.

Gómez Gallego and Gómez García (2017) *Neurologia*	Probable mild to moderate AD (CDR = 1–2) Music intervention group only (*n* = 42)	6-weeks, including two 45 min group sessions per week	Active music therapy Led by 2 music therapists Sessions included a welcome song (patients greeted and introduced themselves), rhythmic accompaniment (clapping hands or playing music instruments), moving to background music (moving arms and legs to music, dance therapy with hoops and balls), guessing songs, and farewell song. Individualized	Measures taken at 3 and 6 weeks. No follow up. *Cognitive:* Mini-Mental Status Examination (MMSE) *Behavioral:* Neuropsychiatric Inventory (NPI) Hospital Anxiety and Depression Scale (HADS) Barthel Index (BI)	*Cognitive:* Significant increase in MMSE scores for orientation, language and memory from beginning to end of sessions. *Behavioral:* Non-significant decrease in total NPI scores (decreased subscores in delusions, hallucinations, agitation, anxiety, apathy, and irritability in moderate dementia). Decreased especially anxiety and depression according to HADS. No change in functional dependence according to BI

Li et al. (2015) *Neuropsych-iatric Disease and Treatment*	Mild AD (CDR = 0.5∼1) *n* = 41 AD participants divided into music intervention group (*n* = 21) vs. control group (*n* = 21)	24-weeks, including seven 1 h sessions per week (divided into 30 min in morning and 30 min at night)	Music listening Listening with caregivers Listen to Mozart’s Sonata for Two Pianos in D major for 30 min in morning and Pachelbel’s Canon in D major for Violins for 30 min before sleep Not individualized	Measures taken at baseline and at 6 months. No follow up. *Cognitive:* Cognitive Abilities Screening Instrument (CASI) Clinical Dementia Rating Scale with sum of box scores (CDR-SB) Mini-Mental State Exam CASI-estimated (MMSE-CE) *Behavioral:* NPI	*Cognitive:* No significant difference in total cognitive scores in MBI vs. control groups. Significant difference found in abstraction, slight improvement of short term memory *Behavioral:* No significant findings

Arroyo-Anlló et al. (2013) *BioMed Research International*	Mild-moderate AD (average MMSE of 19) *n* = 40 AD participants divided into individualized music group (*n* = 20) vs. non-individualized group	12 weeks, including three 2–4 min sessions per week	*Music listening* Listening with caregivers Listen to music attentively at home, in a quiet room with headphones and without distractions Individualized and not individualized groups	Measures then 1–2 weeks before and 1–2 weeks after MBI completion. No Follow up. *Cognitive:* Mini-Mental State Exam (MMSE) F-A-S Test Self-Consciousness Questionnaire	*Cognitive:* No change in cognition for familiar group, although unfamiliar group experienced decline. Significant differences of self-consciousness in familiar music group, specifically personal identity, affective state, moral judgement, and body representation.

Sakamoto et al. (2013) *International Psychogeri-atrics*	Severe AD (CDR = 3) *n* = 39 AD participants divided into music listening group (*n* = 13), active group (*n* = 13) and no intervention group (*n* = 12)	10 weeks, including one 30-min session per week	Active and music listening *Active:* Led by two music therapists, four occupational therapists, and six nurses. Listening to music on a CD player, while doing activities such as clapping, singing, and dancing. *Receptive:* Listening with caregiver Passively listened to the selected music via a CD player Both individualized	Measures taken 2 weeks before study and at MBI completion. Follow up 3 weeks after MBI completion *Behavioral:* Faces Scale Behavioral Pathology in Alzheimer’s Disease Autonomic nerve index [Heart rate (HR) and high-frequency (HF)]	*Behavioral:* Immediately after one session: Increased parasympathetic activity (HR and HF) in receptive and active MBI, as well as improved emotional state (active slightly better) resulting in reduced stress After 10-week MBI completion: decreased anxiety, affective disturbance in receptive group and active group; decreased anxiety, affective disturbance, paranoid/delusion, aggression, activity disturbance and Global Rating in active only, as well as reduced caregiver burden. Post intervention: Effects disappeared after 3 weeks.

Guétin et al. (2009) *Dementia and Geriatric Cognitive Disorders*	Mild-moderate AD (MMSE 12–25) *n* = 30 AD participants, divided into MBI group (*n* = 15) and no MBI group/reading (*n* = 15)	16 weeks, including one 20-min session per week	Music listening Listening to music alone in controlled setting Music streamed to nursing home rooms via headphones Individualized	Measures taken at MBI initiation, 4, 8, and 16 weeks. Follow up 8 weeks after MBI completion *Behavioral:* Hamilton Scale Geriatric Depression Scale	*Behavioral:* Significant decrease in anxiety at 4 weeks and persisted after 2 months; significant decrease in depression after 3 weeks and persisted at 6 months


*AD, Alzheimer’s disease; n, number of participants; MMSE, Mental Status Examination; CT, cognitive training; AMT, Active Music Therapy; NE, Neuro-education; WFT, Word Fluency Task; SST, Short Story Test; RAVLT, Rey Auditory Verbal Learning Test; RCPM, Raven Colored Progressive Matrices; BDI, Beck Depression Inventory; STAI, State Trait Anxiety Inventory; LSNS, Lubben Social Network Scale; CDR, Clinical Dementia Rating; NPI, Neuropsychiatric Inventory; HADS, Hospital Anxiety and Depression Scale; BI, Barthel Index; CASI, Cognitive Abilities Screening Instrument; CDR-SB, CDR-sum of boxes; MMSE-CE, MMSE-CASI estimated; MBI, music-based intervention.*

## Discussion

A diverse method of approaches for intervention were implemented across studies, including varying music selection and method of exposure. In this discussion, we will look at the effects of music selection and intervention approach on cognition or behavior in persons with AD.

Studies which used individualized playlists (Guétin et al., 2009; Arroyo-Anlló et al., 2013; Sakamoto et al., 2013; Gómez Gallego and Gómez García, 2017) resulted in improved outcomes for cognition and behavior in both active music therapy and music listening compared to methods that used experimenter chosen music. For example, Li et al. (2015) selected pre-determined classical music pieces for the participants. The use of classical music in order to enhance cognition is known as the “Mozart Effect” (Rauscher et al., 1993). This phenomenon has been attributed to acute arousal caused by the enjoyment of listening to music, and not because classical music has the ability to enhance cognition beyond the music listening session (Chabris, 1999; Thompson et al., 2001). Li et al. (2015) did not find any changes in behavior correlated to their intervention and found only small changes in cognition when looking into subcategories of cognitive tests. Additionally, Giovagnoli et al. (2017) active music therapy did not engage participants to music that was known to them. The authors found only slight clinical improvements in a verbal initiative executive functioning task and episodic memory (in 7.69 and 23.08% of participants, respectively), but much less so than the cognitive training (CT) condition (61.54 and 38.46%, respectively). This study also found that, although some mood improvements were found in their intervention, the same improvements were found in CT and Neuroeducation (NE) groups, and therefore can be attributed to the creation interpersonal relationships with group members, experience of a change of setting from their regular routine, or interaction between group members and clinicians and music therapists (Raglio and Oasi, 2015) rather than a unique effect of music on mood, behavior, or cognition.

Conversely, our investigation found that intervention approaches which provided individualized music playlists generally found positive outcomes in both cognition and behavior for their participants (Guétin et al., 2009; Arroyo-Anlló et al., 2013; Sakamoto et al., 2013; Gómez Gallego and Gómez García, 2017). Arroyo-Anlló et al. (2013) compared familiar and unfamiliar generalized music listening groups and found improvements in self-consciousness and global cognition in the familiar music group compared to the unfamiliar group. Gómez Gallego and Gómez García (2017) used individualized music in their active intervention and found improvement in orientation, language and memory domains cognitively, as well as improvements in anxiety and depression. Sakamoto et al. (2013) investigated only behavioral outcome measures and found acute improvements in anxiety, affective disturbance, aggression, psychosis, and activity disturbance. Finally, Guétin et al. (2009) found improvement in anxiety and depression in their individualized music listening intervention. The benefit of music on cognition and behavioral symptoms of AD have been commonly attributed to arousal and improved mood (Chabris, 1999; Thompson et al., 2001). However, our investigation showed that music that is individualized to the patient show greater benefits than music that the patient does not know, suggesting more than arousal is involved in improving cognitive and behavioral outcomes for patients. We suggest this is due to the positive effects that long-known music can have on the brain of AD persons. Previous literature suggests that music can evoke autobiographical memories in persons with AD (Foster and Valentine, 2001; Irish et al., 2006; El Haj et al., 2012b), particularly if the music is self-chosen and known to the patient (El Haj et al., 2015). The deterioration of memory in AD is often linked with impairment of autonomy and the sense of Self (Fargeau et al., 2010). Since music that is known to the patient has the ability evoke autobiographical memories (El Haj et al., 2015), this can in turn improve self-consciousness, global cognitive functioning, and neuropsychiatric symptoms in individuals with AD (Arroyo-Anlló et al., 2013).

Our investigation provided evidence that the intervention approach used may also have an effect on cognition and behavior. Namely, Sakamoto et al. (2013) uniquely compared their generalized music listening intervention to an active music therapy group, and found that both methods of treatment showed generally significant results in behavioral symptoms such as anxiety and affective disturbance. However, those who underwent music therapy experienced additional benefits compared to the music listening group in the domains of paranoid/delusion, aggression, activity disturbance and overall rating of behavioral symptoms in persons with more severe AD. However, these results disappeared after the intervention. Conversely, the other two studies utilizing active music therapy provided conflicting results on both cognition and mood (Giovagnoli et al., 2017; Gómez Gallego and Gómez García, 2017), where Giovagnoli et al.’s (2017) active music therapy saw no better improvements on mood than NE or CT and significantly less cognitive outcomes than CT, while Gómez Gallego and Gómez García (2017) found significant cognitive improvement in both cognition and mood. Since Gómez Gallego and Gómez García (2017) used an individualized approach and Giovagnoli et al. (2017) did not, these improvements may be due to type of music used (individualized vs. not individualized) and not the intervention approach (music listening vs. active music therapy).

The music intervention approach may also have different impacts on the sympathetic and parasympathetic nervous systems of participants, which may affect acute and long-term outcomes. Active music therapy tends to focus more on and activity and the social aspects of participation (Raglio and Oasi, 2015), such as interactions between client and clinician and the act of clapping, dancing and playing instruments. While all the active music therapies encouraged interpersonal relationships with others and emotional introspection, the music listening approaches undergone by the studies in this review focused more on engaging participants to music they enjoy and know from their past. Additionally, music listening approaches provided a calm and relaxing environment to induce relaxation, while active music therapies increased arousal with participation (Sakamoto et al., 2013). As postulated by Peck et al. (2016) music can modulate the sympathetic and parasympathic autonomic nervous systems and, as a consequence, physiological responses. During active music therapy sessions, Sakamoto et al. (2013) measured heart rate immediately after active sessions and music listening as part of their protocol and found that heart rate was elevated after active music sessions when compared to the music listening sessions. Therefore, the effects of active sessions may be based more on arousal mechanisms to reduce behavioral symptoms acutely, while music listening may act to train participants in relaxation techniques that provide parasympathetic regulation and prolonged benefits to the patients. Additionally, Guétin et al. (2009) used a specific music listening technique called the “U Sequence” which was developed specifically to gradually relax the listener (Guétin et al., 2005; Jaber et al., 2007). The authors created this type of music program with individualized music for each participant. In addition to providing benefits for anxiety and depression after completion of intervention, the authors found the effects of the therapy lasted 6 months after completion. Of the three studies that followed up with participates after completion of the intervention (Guétin et al., 2009; Sakamoto et al., 2013; Giovagnoli et al., 2017), no other study included in our review found the benefits from their program persisted after termination of the intervention. Our findings of the benefits of music listening for persons with AD compared to active music therapy is supported by a recent review by Tsoi et al. (2018), who also found that music therapies involving music listening provided greater benefits than active music therapy.

Our review has limitations that should be addressed. The aim of this narrative review was to determine the impact of various music intervention approaches specifically for persons with AD. However, the studies included vary in other aspects which may impact the results, such as participant age, disease severity, cognitive level, outcome measures, length of intervention, etc. Furthermore, the methodology differed within musical approaches. For example, music listening regimens ranged in their method of exposing participants to music, such as via headphones or streamed through the room. Music therapy techniques differed in the activities conducted during the sessions. As well, our investigation included only a small amount of studies, which may result in low power of our results.

## Conclusion

In this review, we discussed six studies involving a music intervention approach for AD persons that met our search criteria. In summary, our investigation into the aforementioned studies suggested music interventions which used individualized music playlists and focused on relaxation techniques tended to yield greater benefits on AD persons. We hypothesize this is due the enhancement of autobiographical memory, autonomy, and parasympathetic modulation which in turn has positive effects on cognition and behavior.

While there are many reviews available looking at the effect of music on various symptoms, intervention studies that assess music, cognition, and memory are less common. As cognitive decline is a main effect of AD and can contribute further to increasing neuropsychiatric symptoms, medication use, and visits to the emergency room, investigation of music on cognition in the future is imperative. As well, more rigorous behavioral studies, as well as systematic reviews and meta-analyses, are needed to investigate the impact of individualized vs. not individualized music to make stronger evidence-based conclusions. Lastly, although many studies have investigated outcomes pre- and post-music intervention for AD persons, there is a lack of studies investigating brain changes associated with a music intervention. As well, imaging studies investigating brain areas involved in music listening have thus far only been investigated in healthy, young controls. Such studies could provide empirical evidence to further the understanding of mechanisms involved in musical memory, and how music can work to improve cognition and behavior in persons with AD.

## Author Contributions

ML formulated the research paper idea, wrote the main body of the manuscript, participated in revisions, and submitted the final manuscript. MT provided substantial edits to the paper and final draft, and aided in the interpretation of the paper. LF contributed to the formulation of the research paper idea, provided substantial edits to the paper and the final draft, and aided in the interpretation of the paper. TS provided substantial edits to the paper and final draft, and aided in the interpretation of the paper. JB provided edits to the final draft, and aided in the interpretation of the paper. DM provided edits to the final draft, and aided in the interpretation of the paper. CF contributed to the formulation of the research paper idea, provided substantial edits to the paper and the final draft, and aided in the interpretation of the paper.

## Conflict of Interest Statement

The authors declare that the research was conducted in the absence of any commercial or financial relationships that could be construed as a potential conflict of interest.
